# Ontogenetic and spatial variation in the trophic ecology of the shortfin mako shark (*Isurus oxyrinchus*) in temperate eastern Australia

**DOI:** 10.1111/jfb.70667

**Published:** 2026-09-27

**Authors:** Maëlle Devillebichot, Jane E. Williamson, Julian G. Pepperell, Tristan A. Guillemin

**Affiliations:** ^1^ School of Natural Sciences Macquarie University, Wallumattagal Campus Sydney New South Wales Australia; ^2^ Pepperell Research and Consulting Pty Ltd Noosaville Queensland Australia

**Keywords:** Eastern Australia, fisheries management, ontogenetic shifts, shortfin mako shark, stable isotopes, trophic niche

## Abstract

The shortfin mako (*Isurus oxyrinchus*) is a fast‐swimming pelagic shark of global ecological and socioeconomic importance, yet it is relatively understudied in temperate eastern Australian waters. This study investigated the trophic ecology of mako sharks using stable isotope analysis (SIA) of nitrogen (δ^15^N), carbon (δ^13^C) and sulphur (δ^34^S). White muscle tissue samples were obtained from captured during game fishing tournaments along the New South Wales coast between 2005 and 2025. Ontogenetic shifts in isotopic composition were observed across individuals. δ^15^N values decreased with increasing body size, while δ^13^C values increased with size, suggesting that larger sharks may feed more frequently on prey of lower trophic positions. In contrast, δ^34^S values indicated mainly pelagic foraging across all size classes. Isotopic niche models revealed partial niche overlaps between size groups, with convergence in larger individuals, indicating shared resource use at maturity. Seasonal effects were weak, potentially reflecting sampling bias and the slow isotopic turnover rate of shark muscle. At the population level, their ecological role appears as a generalist, although individual specialization may occur. These findings demonstrate that ontogenetic variation and spatially variable foraging behaviour should be considered in fisheries management. In particular, the apparent reliance of larger individuals on coastal prey suggests that they may be more vulnerable to recreational fisheries than previously assumed. This study provides the first sulphur‐inclusive stable isotope analysis of *I. oxyrinchus* and offers a species‐specific trophic perspective for temperate eastern Australia. It highlights the importance of integrating stable isotope data with tagging studies, prey sampling and stomach content analyses to better resolve trophic pathways. Such integrative approaches will support ecosystem‐based fisheries management, help reduce bycatch risks, and strengthen conservation strategies for this endangered species.

## 1 INTRODUCTION

As apex or mesopredators, many large shark species occupy top trophic levels and exert strong ecological influence within marine food webs (Ferreira et al., 2017; Velasco‐Tarelo et al., 2024). Through top‐down control, they contribute to ecosystem stability and health and help link distant habitats through wide‐ranging movements (Heupel et al., 2014). Consequently, declines in shark populations can trigger trophic cascades and disrupt marine ecosystem functioning (Baum & Worm, 2009). Despite their ecological importance, sharks are increasingly threatened by overexploitation from commercial and recreational fisheries, both as target species and bycatch (Davidson et al., 2022). Improving our understanding of shark trophic ecology is therefore critical for developing effective conservation strategies and supporting the transition towards ecosystem‐based fisheries management (Polo‐Silva & Bustamante, 2024; Shiffman et al., 2012).

A key component of shark trophic ecology is understanding how trophic roles vary among individuals and life stages. Many species undergo ontogenetic dietary shifts as growth and energy demands change, influencing habitat use, prey selection and ecological function within food webs (Raoult et al., 2019; Shiffman et al., 2012; Velasco‐Tarelo et al., 2024). Although ontogenetic changes are a major driver of trophic variation, migration and habitat shifts can also alter prey composition and trophic position by exposing individuals to different prey communities and foraging opportunities (Ikpe, 2025; Lopez et al., 2012). For example, tiger sharks and great hammerheads consume larger prey as they increase in size (Ferreira et al., 2017; Rosas‐Luis et al., 2016), whereas white sharks in the northeastern Pacific exhibit a decrease in trophic level with increasing size, reflecting shifts in habitat rather than prey size (Carlisle et al., 2012). Incorporating these ontogenetic differences is essential for accurately evaluating shark ecological roles and informing management and conservation strategies (Hussey et al., 2012).

Stable isotope analysis (SIA) is widely used to investigate shark feeding ecology and trophic structure (Klarian et al., 2018; Malpica‐Cruz et al., 2013; Revill et al., 2009). This technique has been widely adopted because muscle tissue can be obtained through minimally invasive biopsy sampling while providing cost‐effective, longer‐term dietary estimates (over periods of several months to several years) compared with stomach content analyses, which typically reflect only recent feeding (Niella et al., 2021; Polo‐Silva & Bustamante, 2024; Shiffman et al., 2012). The two primary stable isotope ratios used as dietary tracers are nitrogen (δ^15^N = ^15^N/^14^N) and carbon (δ^13^C = ^13^C/^12^C) (Abrantes & Sheaves, 2024; DeNiro & Epstein, 1978; DeNiro & Epstein, 1981). δ^15^N values are commonly used to estimate relative trophic position of consumers, as they generally increase with each trophic transfer, whereas δ^13^C values provide information on the origin of dietary carbon and can help identify differences in foraging habitats and energy pathways within food webs (Estrada et al., 2003; MacNeil et al., 2005; Post, 2002). More recently, sulphur stable isotope ratios (δ^34^S = ^34^S/^32^S) have been used to distinguish pelagic and benthic energy sources, providing an additional dimension to trophic studies (Raoult et al., 2019; Thomas & Cahoon, 1993; Wells et al., 2008). Although shortfin mako sharks are primarily pelagic predators, δ^34^S values can provide complementary information on the contribution of different basal energy sources supporting their prey, refining interpretations of their trophic ecology. Despite its potential, δ^34^S values have yet to be applied to investigate the trophic ecology of the shortfin mako shark, representing an opportunity to refine understanding of its feeding dynamics.

The shortfin mako shark (*Isurus oxyrinchus* Rafinesque, 1810) is a fast‐swimming pelagic predator with a global distribution across tropical and temperate waters (Maia et al., 2006; TSSC, 2014). This species has a high trophic position among pelagic sharks (Revill et al., 2009), therefore, playing an important role in regulating food webs through top‐down control across pelagic, coastal and oceanic ecosystems (Corrigan et al., 2018; Rosas‐Luis et al., 2016). In the open ocean, *I. oxyrinchus* typically exhibits diel vertical migrations, spending most of its time in near‐surface waters, with occasional deeper dives during the daytime (Andrzejaczek et al., 2022; Lin et al., 2026; Vaudo et al., 2016); this behaviour coincides with its prey preferences, which are dominated by teleost fish and cephalopods (Calle‐Morán et al., 2023). Shortfin makos are also highly migratory, moving over large spatial scales. Juveniles often show greater fidelity to coastal habitats on continental shelves, whereas adults undertake broader offshore movements, alternating between periods of coastal residency and long‐distance oceanic transit (Chan, 2001; Nasby‐Lucas et al., 2019).

In Australian waters, *I. oxyrinchus* occurs along most of the coastline, showing preference for continental shelf‐break waters (Green et al., 2024; TSSC, 2014). Tagging studies reveal that makos tagged along the New South Wales coast generally remain within the Southwest Pacific, with movements extending to New Zealand, New Caledonia and the Solomon Islands (Chan, 2001; Corrigan et al., 2018). Genetic evidence also suggests the presence of a distinct Southwest Pacific population, highlighting the need for region‐specific management strategies (Corrigan et al., 2018). Despite this, the first regional stock assessment (Large et al., 2022) identified significant data deficiencies and model instability, underlining the urgent need for more ecological and biological data.

Shortfin makos experience substantial fishing pressure in temperate eastern Australia. Despite being listed as “Endangered” globally (Rigby et al., 2019) and “Vulnerable” in the Indo‐West Pacific (Cailliet et al., 2009), they remain a common bycatch species in the Eastern Tuna and Billfish Fishery and are also targeted in recreational fisheries (Ghosn et al., 2015; Rogers et al., 2015). Their highly migratory behaviour further complicates management because individuals regularly cross international boundaries, while recent tagging data indicate declining numbers of tagged makos, suggesting reduced fishing effort, population change or both (DPIRD, 2023; Robertson & McIntyre, 2019).

Globally, isotope studies indicate that shortfin mako sharks occupy a broad trophic spectrum and use several habitats throughout ontogeny, including coastal, oceanic, pelagic and benthic environments (Velasco‐Tarelo et al., 2024). Larger individuals often display higher δ^15^N values, suggesting elevated trophic positions and broader diets (Malpica‐Cruz et al., 2013). Understanding these patterns in Australian waters is particularly relevant given regional differences in oceanography, prey availability and fishing pressure.

Together, these factors highlight the need to better characterize the trophic ecology of shortfin mako sharks in temperate eastern Australia to inform conservation and management. This study used stable isotopes of nitrogen (δ^15^N), carbon (δ^13^C) and sulphur (δ^34^S) to investigate the trophic ecology of *I. oxyrinchus* and how it varies through ontogeny. Given the documented ontogenetic shifts in trophic position and habitat use, as well as anecdotal observations from the recreational fishery suggesting that smaller individuals are more frequently caught closer to the coast, while larger individuals are caught further offshore, we hypothesized that larger individuals would occupy higher trophic positions (higher δ^15^N values) and rely more on offshore pelagic food webs (lower δ^13^C values), whereas smaller individuals would feed more in coastal habitats. Considering the predominantly pelagic ecology of shortfin makos, we further expected relatively enriched high δ^34^S values. By combining these tracers, this study contributes to a more comprehensive understanding of the trophic role of shortfin makos and provides new insights for their management in Australian waters.

## 2 MATERIALS AND METHODS

### 2.1 Sample collection

White muscle tissue samples from shortfin mako sharks were collected during game fishing tournaments held by clubs affiliated with the New South Wales Game Fishing Association (GFA) in Australia (Ghosn et al., 2015; Pepperell, 2024). Sampling took place at 11 locations along the east Australian coastline between Port Stephens (32.717° S and 152.105° E) and Bermagui (36.426° S and 150.073° E) between May 2005 and May 2025. Samples (approximately 8–12 cm^3^) containing both skin and underlying muscle were excised from the ventral surface of each individual, adjacent to the anal fin. At the tournament port, each shark was sexed, weighed on certified scales by the tournament weighmaster, and its fork length (FL) was recorded to the nearest cm. After collection, all tissue samples were brought back to the laboratory, frozen at −30°C and stored under these conditions until further processing to ensure tissue integrity. Some samples had been stored at the processing laboratory of the Department of Biological Sciences of Macquarie University, Sydney. Additional muscle samples were obtained from the archived collection of Pepperell Research & Consulting, which were held in a −80°C freezer at the premises of the Ocean Health Foundation, Coolum, Queensland and subsequently transported frozen to Macquarie University.

### 2.2 Stable isotope analyses

White muscle tissue samples (without skin) were first dried in a ventilated oven (Binder ED115) set at 60°C for 48 h, then pulverized into a fine powder using a ball mill (Retsch Mixer Mill MM400) operating at 30 Hz for 30–60 s. Lipids and urea were then extracted from the samples; extraction was preferred over mathematical isotope correction models due to potential prediction errors, making it less suitable for universal application (De Lecea & De Charmoy, 2015). Lipid extraction was performed following the established method of Bligh and Dyer (1959) with variations from Burke et al. (2024). Each powdered sample was immersed in five successive 5 mL volumes of a 2:1 chloroform: methanol solvent mixture. After each immersion, samples were centrifuged for 5 min at 3000 rpm to separate the solvent phase. This sequence was repeated three consecutive times to ensure thorough lipid removal. Immediately after lipid extraction, urea extraction was carried out. Samples were immersed in five successive 5 mL volumes of deionized water, followed by centrifugation for 5 min at 3000 rpm and decanting of the supernatant, again repeating the process three times consecutively to maximize urea removal (Burke et al., 2024). Finally, treated samples were returned to the oven and dried once more at 60°C for at least 48 h to eliminate any remaining solvent or moisture. Resulting powdered tissues were placed in separate 5 mL sterile Techno‐Plas tubes and sent to Griffith University Stable Isotope Laboratory (Nathan Campus, Queensland, Australia). Samples were further analysed using an Isotope Ratio Mass Spectrometer (IMRS Thermo Fisher Scientific, model Delta V Advantage) coupled to an Elemental Analyser (Thermo Fisher Scientific Flash 2000).

Isotope signatures are measured as deviations from an international reference standard and expressed as a delta (δ) value in parts per 1000 (‰) (Boecklen et al., 2011; Peterson & Fry, 1987). The international reference standards used for δ^13^C, δ^15^N and δ^34^S were Pee Dee belemnite, atmospheric nitrogen and Vienna‐Canyon Diablo Troilite, respectively. Isotope signatures were calculated using the formula: δ^H^X = [(*R*_sample_/*R*_standard_) – 1] * 1000, where X is the element of interest, H is the heavy isotope mass of the element and *R* is the corresponding ratio of heavy to light isotope (Carlisle et al., 2012). Samples and standards were prepared with vanadium pentoxide to assist complete combustion at 1020°C under oxygen addition. Results were calibrated against primary standards: IAEA‐N1 and IAEA‐N2 for δ^15^N, IAEA‐CH‐6 for δ^13^C and IAEA‐S1, IAEA‐S2 and IAEA‐S3 for δ^34^S. Secondary standards calibrated against these primary ones were analysed in each tray to monitor instrument calibration and precision: glycine (δ^13^C = −34.0‰, δ^15^N = 10.8‰) and potassium sulphate (δ^34^S = 10.8‰), alongside NBS‐127 for sulphur and NIST SRM 1577c Bovine liver (elemental standard for N and S). Mean measured values ± SD for the internal standard (Glycine LSU‐1 Delta, *n* = 12 per run) were − 34.00 ± 0.18‰ for δ^13^C, 10.71 ± 0.18‰ for δ^15^N and 10.80 ± 0.43‰ for δ^34^S.

### 2.3 Statistical analyses

All statistical analyses were performed in R v4.3.2, with statistical significance set at *p* < 0.05 (R Core Team, 2023). All data were assessed for normality and homogeneity of variances prior to analysis. When one or both assumptions were violated, non‐parametric tests were applied: Mann–Whitney U test for pairwise comparisons and Kruskal–Wallis test followed by post hoc Dunn's tests (Bonferroni correction) for multiple comparisons. To examine potential ontogenetic shifts in diet and foraging ecology, individuals were assigned to three FL classes: ≤200 cm, 201–250 cm and >250 cm. These sizes were selected to reflect growth stages related to sexual maturity in *I. oxyrinchus* that may be associated with shifts in resource and habitat use. These sizes were selected to broadly reflect growth stages related to sexual maturity in *I. oxyrinchus* (estimated at 179 cm for males and 258 cm for females; Stevens, 1983) that may be associated with shifts in resource and habitat use. Thresholds were slightly adjusted (using 200 and 250 cm rather than 179 and 258 cm) to achieve a more balanced distribution of individuals across groups and maximize statistical power. Given that size‐at‐maturity differs between sexes, potential sex‐based differences were evaluated prior to grouping; however, sex had no significant effect on isotopic values, justifying the pooling of males and females. Linear regressions were used to determine the relationship between FL and the three isotope ratios. In addition, univariate linear models were performed to observe the combined effect of size, sex and season on each isotope ratio using the lm function. Model residuals were all tested for normality and homogeneity to verify assumptions. Results were obtained via Type II analysis of variance (ANOVA) using the anova function from the lmertest package and Tukey's post hoc tests to detect pairwise differences. Location and year were excluded due to a lack of consistent coverage.

Isotopic niche metrics were calculated by size classes, and bivariate niche spaces for isotope pairs were graphically represented using the Stable Isotopic Bayesian Ellipse method (SIBER package) (Jackson & Parnell, 2023). For this study, we used size‐corrected Bayesian standard ellipse areas (SEA_c_), which are less sensitive to small sample sizes. Core isotopic niches were defined as 40% SEA_c_ ellipses, providing a conservative estimate and reducing the influence of outlier values, while 95% ellipses were used to visually represent the total trophic niche. Pairwise niche overlaps were expressed as the percentage of shared SEA_c_ and were reported for both core (40%) and total (95% ellipses) and calculated by dividing the area of overlap by the combined SEA_c_ of the two groups (Wang et al., 2022). To integrate all three isotope ratios simultaneously, Bayesian standard ellipse volumes (SEV_b_) were estimated for each size class using the nicheROVER package (Lysy et al., 2023). It generated directional estimates of niche overlap (the probability of overlap of one niche into another) from three‐dimensional isotopic niches. Overlaps were primarily calculated using 40% SEV_b_ ellipses, representing the core isotopic niche, based on 1000 posterior simulations for each group with a probability level of 95% and were reported as mean values with 95% credible intervals.

## 3 RESULTS

### 3.1 Effect of size and season on isotope values

Muscle tissue from 108 shortfin mako sharks was collected but only 94 individuals were appropriate for analysis, because 13 animals had no recorded sex and/or FL, and another individual had an abnormally out‐of‐range isotope value (δ^15^N > 25‰), which was considered an outlier. FL was chosen over total length because it was recorded for a larger number of individuals. The final dataset consisted of 56 females and 38 males, with a mean FL of 224 ± 42 cm (range 169–349 cm). Sampling covered multiple years and locations across NSW (Table 1).

**TABLE 1:** Number of muscle samples (one per individual) collected at each location, with year of collection and sex differentiation.

Sampling location (year)	Sex		
	♂	♀	NR
Port Stephens (2009–2012, 2014–2018, 2020–2022, 2024–2025)	7	20	2
Newcastle (2023)	0	0	1
Lake Macquarie (2009–2010, 2021–2022, 2025)	8	0	0
Broken Bay (2012, 2024)	1	1	0
Watson's Bay (2013, 2019–2022, 2024)	4	6	4
Botany Bay (2012, 2020)	1	2	0
Port Hacking (2011, 2023–2025)	1	6	0
Wollongong (2010–2012, 2020)	25	11	0
Kiama (2012–2013)	0	2	0
Jervis Bay (2020)	0	3	0
Bermagui (2005)	1	0	0
NR	0	2	0

Abbreviation: NR, not recorded.

Mean values of δ^15^N, δ^13^C and δ^34^S were 15.1 ± 0.7‰, −16.2 ± 0.7‰ and 18.9 ± 1.0‰, respectively. To examine potential ontogenetic shifts in diet and foraging ecology, individuals were assigned to one of three FL classes: ≤200 cm, 201–250 cm and >250 cm. δ^15^N values ranged from 13.3 to 16.9‰, δ^13^C from −17.5 to −12.8‰ and δ^34^S from 15.5 to 22.5‰ (Table 2). Smaller shortfin makos showed the highest δ^15^N and lowest δ^13^C values, with a gradual decrease in δ^15^N and increase in δ^13^C with FL increasing. δ^34^S values were more variable, without showing any clear size‐related trend (Table 2; Figure 1).

**TABLE 2:** Mean (μ), standard deviation (sd), minimum (min) and maximum (max) values for isotopes δ^15^N, δ^13^C, δ^34^S for the three size classes of shortfin mako sharks sampled. Values are expressed in parts per 1000 (‰). All the NR values for the variables sex and fork length (FL) were removed from the dataset.

FL (cm)	n	δ^15^N	δ^13^C	δ^34^S
≤200	33	15.6 ± 0.7 (13.6–16.9)	−16.6 ± 0.5 (−17.5 to −15.6)	19.0 ± 1.0 (17.2–21.6)
201–250	37	15.0 ± 0.6 (14.0–16.0)	−16.1 ± 0.7 (−17.0 to −12.8)	18.8 ± 1.0 (17.2–22.5)
>250	18	14.7 ± 0.6 (13.3–16.1)	−15.7 ± 0.7 (−16.9 to −13.5)	18.7 ± 1.0 (15.5–20.1)
Total	94	15.1 ± 0.7 (13.3–16.9)	−16.2 ± 0.7 (−17.5 to −12.8)	18.9 ± 1.0 (15.5–22.5)

**FIGURE 1:**
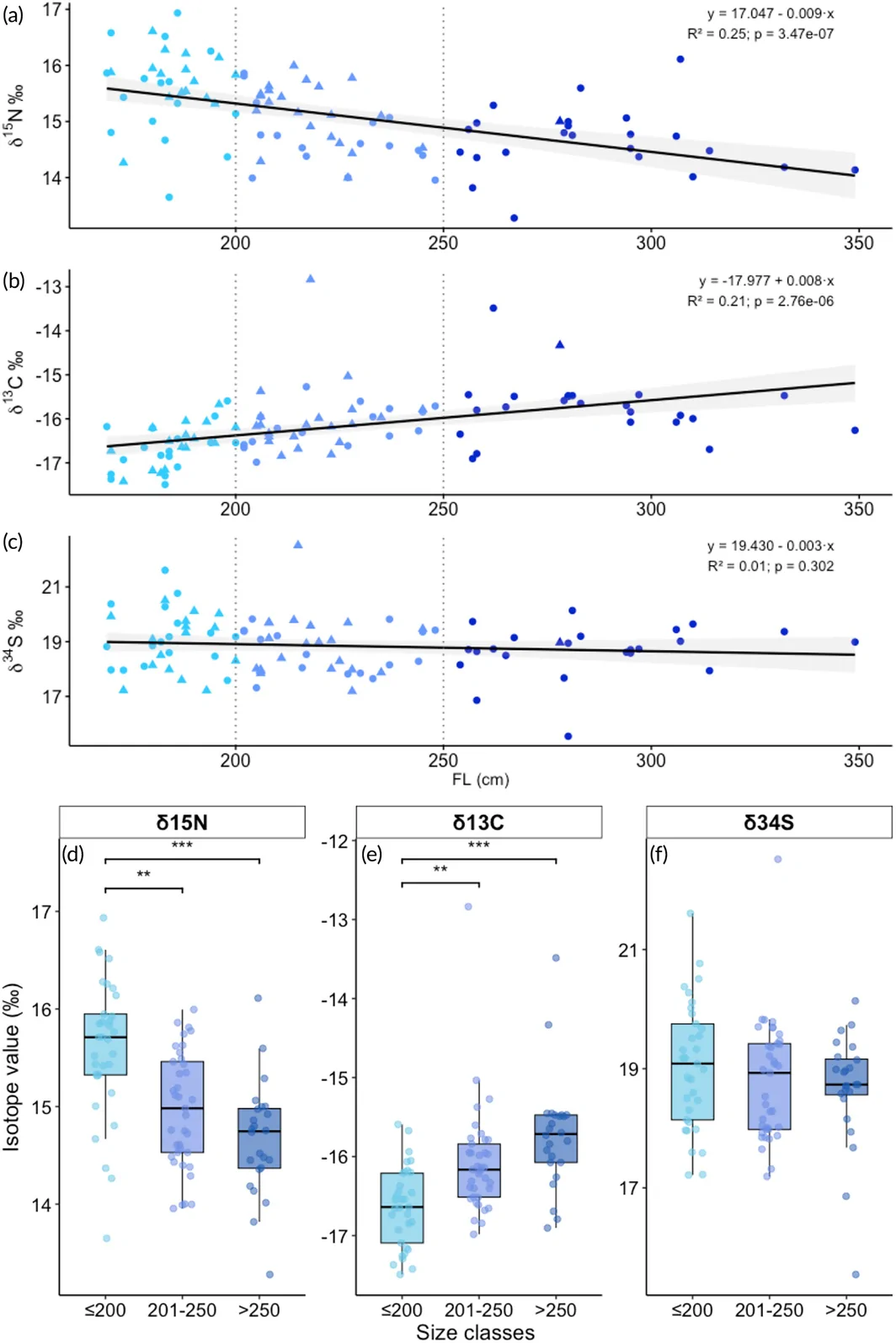
Shortfin mako shark isotopic values δ^15^N, δ^13^C and δ^34^S by FL (a–c) and size category (d–f). Light blue: ≤200 cm; medium blue: 201–150 cm; dark blue: >250 cm. Bar: median; box: 25th‐75th percentile; dots: outliers.

Linear regression revealed a weak but significant negative relationship between FL and δ^15^N values (*R* ^2^ = 0.25, *p* < 0.001) and a weak positive one with δ^13^C values (*R* ^2^ = 0.21, *p* < 0.001) (Figure 1a–c). No significant relationship was found between FL and δ^34^S values (*R* ^2^ = 0.01, *p* = 0.302). When isotope ratios were compared among the three size categories, the Kruskal–Wallis test revealed that both δ^15^N (χ^2^ = 25.1, df = 2, *p* < 0.001) and δ^13^C values (χ^2^ = 27.8, df = 2, *p* < 0.001) differed between groups, whereas δ^34^S did not (*p* = 0.45) (Figure 1d–f). Post hoc Dunn tests (Bonferroni adjustment) indicated that δ^15^N values were significantly higher for individuals in ≤200 cm compared to the other two classes (*p* < 0.01 for 201–250 cm, *p* < 0.001 for >250 cm). δ^13^C was lower in the ≤200 cm category than in 201–250 cm (*p* < 0.01) and >250 cm (*p* < 0.001).

Female shortfin makos were, on average, significantly larger than males (Wilcoxon rank‐sum test, *p* < 0.01), with mean FL of 237 ± 48 cm and 206 ± 22 cm, respectively. Given this size difference, isotopic comparisons between sexes were tested while accounting for FL, in univariate linear models with the isotopic ratios as response variables, sex as a fixed factor (two levels) and FL as a continuous variable (Figure 2). When controlling for size, the ANOVA type II indicated that sex had no significant effect on δ^15^N (df = 1, F1, 91 = 2.86, *p* = 0.09), δ^13^C (df = 1, F1, 91 = 3.15, *p* = 0.08) or δ^34^S (df = 1, F1, 91 = 0.003, *p* = 0.96) values, although there was a weak trend for both δ^15^N and δ^13^C values, with females having lower adjusted isotopic values than males.

**FIGURE 2:**
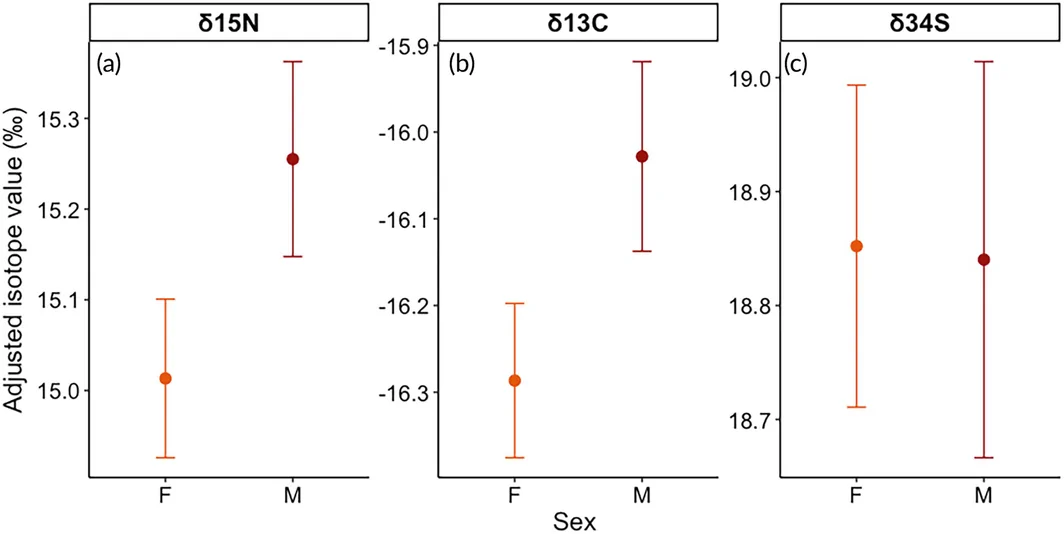
Estimated marginal means (± SE) of δ^15^N (a), δ^13^C (b) and δ^34^S (c) in muscle tissue of female (f) and male (M) shortfin mako sharks, adjusted for FL using univariate linear models. Orange = F, red = M.

The effect of sampling season on δ^15^N, δ^13^C and δ^34^S was tested independently. As only two individuals were sampled during winter months, winter was grouped with autumn to form an “autumn‐winter” category. The resulting groups contained 23 (autumn‐winter), 41 (spring) and 28 (summer) individuals (Figure 3). δ^15^N values were significantly higher in spring than during both autumn‐winter and summer (Kruskal–Wallis, χ^2^ = 20.51, df = 2, *p* < 0.001; Dunn's test, *p* < 0.001 for both). δ^13^C values were higher in summer than in spring (Kruskal–Wallis, *p* < 0.001; Dunn's test, *p* < 0.001) and no seasonal variation was detected for δ^34^S values.

**FIGURE 3:**
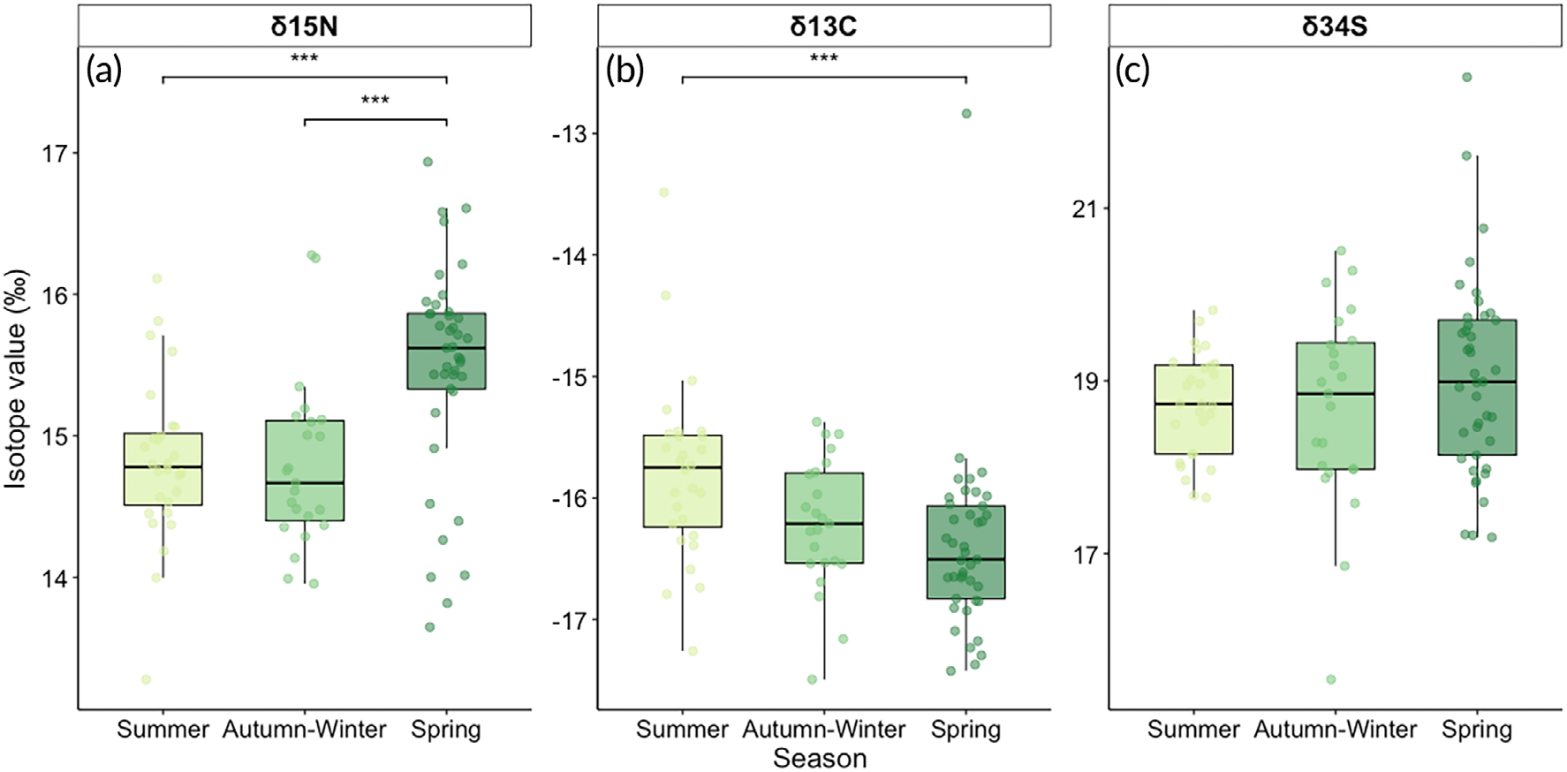
Seasonal variation in isotope values, with seasons representing when the shark's white muscle tissue was sampled. Bar: median; box: 25th‐75th percentile; dots: Individual values. Significance levels from Dunn's post hoc tests are indicated: *** for *p* < 0.001.

To account for the combined influence of size, sex and sampling season on isotope values, univariate linear models were performed with FL, sex and season as fixed effects (Table S1). These models confirmed that FL was the dominant driver of isotopic variation, with ANOVA showing significant effects on δ^15^N (*F*_1,87_ = 11.31, *p* = 0.001) and δ^13^C (*F*_1,87_ = 17.21, *p* < 0.001) values. Sex also had a significant effect on δ^13^C values (*F*_1,87_ = 5.93, *p* = 0.02), with males showing slightly higher adjusted values than females. Sampling season had a significant effect on δ^15^N values, with spring having higher δ^15^N values than autumn‐winter (Tukey's honestly significant difference [HSD]: *p* = 0.03). Season explained a small portion of the variation in δ^13^C values (*F*_2,87_ = 3.30, *p* = 0.04); however, no pairwise seasonal contrast remained significant after Tukey adjustment, indicating a weak seasonal signal. No predictor significantly explained δ^34^S values.

### 3.2 Isotopic niche analysis

We examined the estimated isotopic niches of shortfin mako sharks using Standard Ellipse Areas corrected for small sample size (SEA_c_) across three isotope ratio pairs: δ^13^C‐δ^15^N, δ^13^C‐δ^34^S and δ^34^S‐δ^15^N (Figure 4). The ellipses representing core isotopic niches (40% of the data) showed an important overlap across size classes, with the exception of the ≤200‐cm and >250‐cm categories for the pair δ^13^C‐δ^15^N (Figure 4a). This overlap seemed to increase considerably when considering the full range of isotope values (95% of data).

**FIGURE 4:**
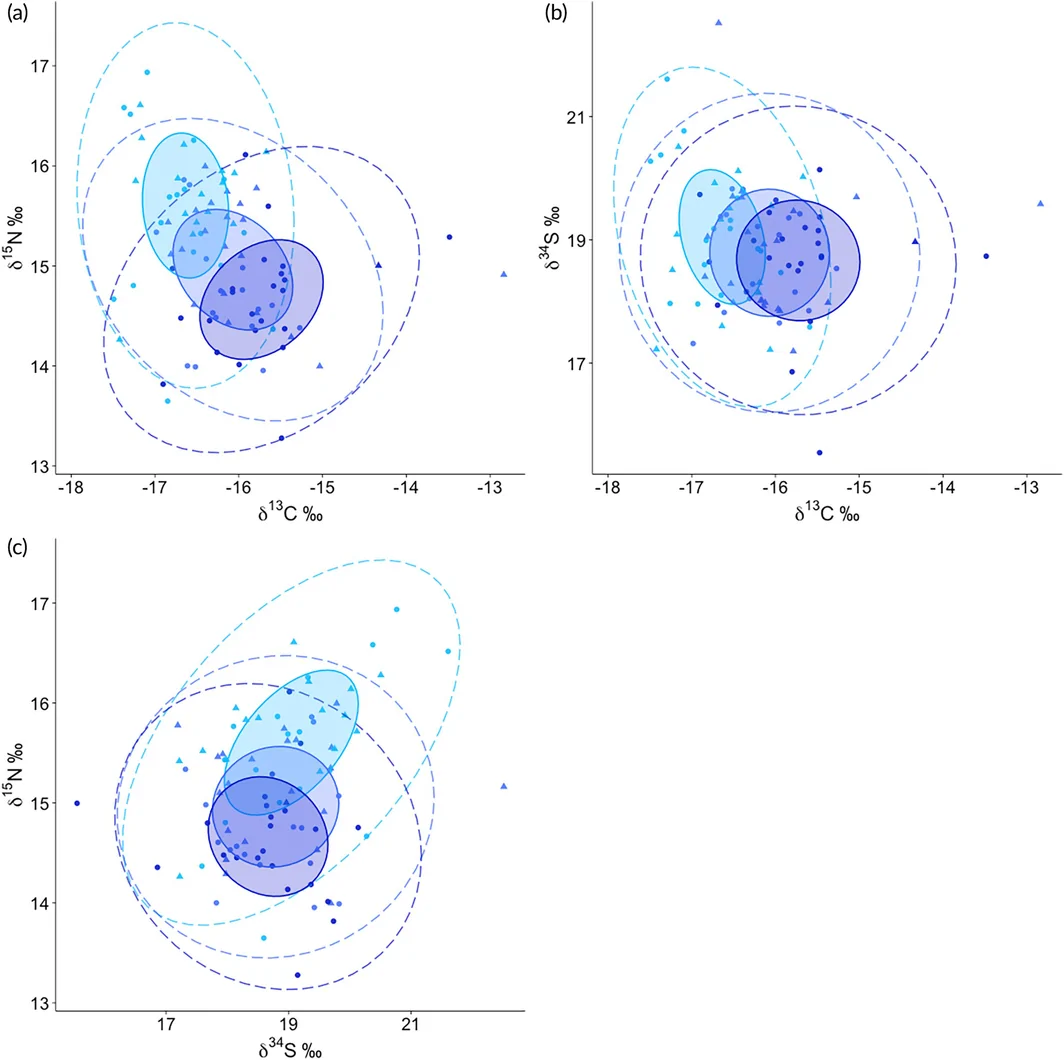
Standard Ellipse Areas corrected for sample size (SEA_c_) per size class for the δ13C‐δ15N pair (a), δ13C‐δ34S pair (b) and δ34S‐δ15N pair (c). Points are isotopic values of each sample, filled ellipses represent the core niche areas (40% of data) and dashed ellipses represent the total trophic niche areas (95% of data). Light blue: ≤200 cm; medium blue: 201–150 cm; dark blue: >250 cm.

Total isotopic niche width of shortfin makos was 1.63‰^2^ for δ^13^C‐δ^15^N, 2.36‰^2^ for δ^13^C‐δ^34^S and 2.31‰^2^ for δ^34^S–δ^15^N (Table S2). Bayesian SEA (SEA_b_) distributions with credible intervals showed generally consistent niche widths across size classes, though patterns varied slightly among isotope pairs (Figure S1). For δ^13^C‐δ^15^N, size classes had very similar niche widths, with SEA_c_ values ranging from 1.16 to 1.33‰^2^. For δ^13^C‐δ^34^S, SEA_c_ values were broadly similar, between 1.69 and 2.30‰^2^, with a subtle difference of the intermediate size class (201–250 cm) having a slightly smaller SEA_c_ (Figure 4b). δ^34^S‐δ^15^N niches were relatively consistent across groups, with SEA_c_ values ranging from 1.82 to 2.10‰^2^ (Figure 4c). All SEA_c_ values are visible in Table S2.

Patterns of niche overlap reflected these trends (Table 3). The largest size classes (201–250 and >250 cm) displayed greater niche similarity, with several overlaps exceeding 50%, whereas the smallest size class (≤200 cm) showed lower overlap with the sharks larger than 250 cm in FL.

**TABLE 3:** Pairwise overlap percentages between isotopic niches of shortfin mako sharks across classes, expressed as a percentage of shared SEA_c_. Values outside brackets correspond to core niches (ellipses covering 40% of the data), while values in brackets represent total niche space (ellipses covering 95% of the data).

		Isotopic pair		
		δ^13^C‐ δ^15^N	δ^13^C‐ δ^34^S	δ^34^S‐ δ^15^N
Size class niche overlap	≤200 and 201–250	53.6 (76.2)	68.9 (86.7)	67.2 (86.2)
	≤200 and >250	0.0 (43.3)	15.5 (61.7)	17.1 (62.0)
	201–250 and >250	27.1 (67.8)	42.7 (73.6)	40.3 (73.2)

A complementary Bayesian analysis was performed to examine the isotopic niches of shortfin mako sharks in three‐dimensional (3D) isotopic space, incorporating δ^15^N, δ^13^C and δ^34^S values. This approach provided an estimation of Standard Ellipse Volumes (SEV_b_), and a better visualization of overlap probabilities between the three size classes. SEV_b_ values ranged from 1.23‰^3^ in the ≤200 cm group to 1.61‰^3^ in the 201–250 group, with an intermediate SEV_b_ of 1.44‰^3^ for individuals >250 cm (Table S3). When all individuals were pooled, the total isotopic niche volume was 2.07‰^3^, reflecting the broader overall trophic niche of the population.

Posterior overlap probabilities differed substantially among the different size categories (Figure 5, Table S3). They varied between 7.0 and 31.4%, with a higher overlap observed among adjacent classes. The ≤200 cm class showed lower overlap probability with the other size categories, and the lowest was observed between the ≤200 cm and >250 cm classes (less than 10%). The highest overlap probabilities were found between the two largest groups, with values exceeding 24% in both directions.

**FIGURE 5:**
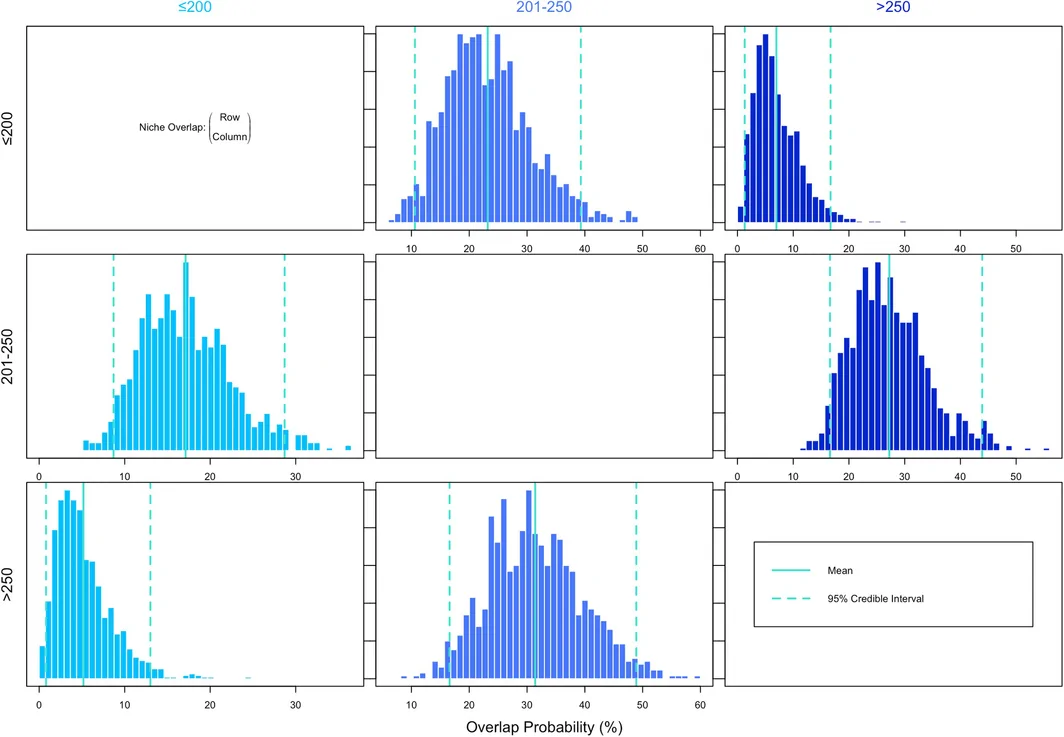
Bayesian Standard Ellipse Volume (SEV_b_) posterior overlap probabilities (probability of the niche of one size class to be found in the niche of another size class). Overlap probability is shown of the size classes displayed in rows onto those displayed in columns. Means and 95% credible intervals are displayed in turquoise.

## 4 DISCUSSION

This study investigated the trophic ecology of shortfin mako sharks in temperate eastern Australia using SIA of δ^15^N, δ^13^C and δ^34^S. We examined ontogenetic shifts in habitat use, trophic level and diet. To our knowledge, this is the first isotopic study specifically targeting *I. oxyrinchus* in temperate eastern Australian waters and the first to incorporate sulphur isotopes in the species. Several ontogenetic patterns were observed; δ^15^N values decreased with animal size while δ^13^C increased. Females were significantly larger than males, but no sex differences were observed once body size was accounted for. Seasonal variation was detected, although interpretation is limited by sampling biases and unknown turnover rates in shark white muscle in this species. Isotopic niche widths were small and similar across size classes, with no or limited overlap between the smallest and the largest individuals, with low probabilities of overlap of one niche onto the other. This overlap increased for individuals over 200 cm FL.

### 4.1 Ontogenetic shift in isotopic values

Mean δ^15^N values were similar to those reported for shortfin makos and other predators in Australian waters, where makos occupied the highest trophic position (Revill et al., 2009). For example, δ^15^N values of 12.5‰ have been reported for blue marlin (Guillemin et al., 2022), while blue sharks had δ^15^N values of 13.6‰ (Revill et al., 2009). However, the decline in δ^15^N values with size contrasts with the pattern typically expected in large predatory sharks, where larger individuals feed at higher trophic levels due to their greater hunting and physiological capacity (Priester et al., 2024; Revill et al., 2009; Tamburin et al., 2019). Because δ^15^N values reflect trophic position relative to diet, larger body size generally corresponds to higher δ^15^N values (Borrel et al., 2010).

Elsewhere, shortfin makos exhibit increasing δ^15^N values with size, including in the Southeastern Pacific (Klarian et al., 2018) and Baja California, where juveniles exhibited higher values than young‐of‐year (YOY) (Velasco‐Tarelo et al., 2024). Several mechanisms may explain the opposite trend observed here. Larger makos off Southeastern Australia may target slightly lower trophic prey such as teleosts rather than cephalopods, other sharks or marine mammals (Carlisle et al., 2012; Malpica‐Cruz et al., 2013). Alternatively, ontogenetic movements into regions with lower prey isotopic baselines could produce lower δ^15^N values without dietary change (Abrantes & Barnett, 2011). A third hypothesis is that smaller shortfin makos may scavenge on large dead prey with high δ^15^N, although this behaviour has never been observed in this species. It is unlikely that maternal signature influenced the stable isotope values observed in the sampled mako sharks, as the smallest individual (169 cm FL) would have been at least 5 years old (Natanson et al. 2006), exceeding the age and size at which maternal effects have been reported to persist (Niella et al., 2021; Olin et al., 2011; Velasco‐Tarelo et al., 2024).

In our study, mean δ^15^N values declined by ~0.9‰ between the smallest and largest size classes. Comparable ontogenetic shifts elsewhere include a 1.5‰ difference in mean δ^15^N values between YOY and older individuals in the eastern Pacific (Velasco‐Tarelo et al., 2024) and increases of 2–3‰ in white sharks (*Carcharodon carcharias*) in the northeastern Pacific (Malpica‐Cruz et al., 2013) and 2.3‰ in tope (*Galeorhinus galeus*) in the Azores between YOY and adults (Priester et al., 2024). While true ontogenetic diet shifts are common in elasmobranchs, baseline isotopic variation driven by regional oceanography may also contribute to the observed pattern. In eastern Australian waters, dynamics associated with the East Australian Current drive considerable spatial and interannual variability in nutrient transport and isotopic baselines (Pattiaratchi & Siji, 2020). Broad‐scale shifts in South Pacific western boundary current transport and climate modes are well known to induce temporal variations in regional isotope baselines through changes in nutrient sources and ocean mixing (Chen et al., 2023). Although temporal baseline shifts may influence year‐to‐year isotopic comparisons, our sample sizes for individual size classes within specific years were insufficient to separate oceanographic drivers from natural biological variability. Further sampling across both early life stages and multi‐year cycles is required to fully resolve baseline variability from true ontogenetic shifts.

In contrast, δ^13^C values increased with body size. Higher δ^13^C values are typically associated with more coastal or inshore benthic food webs, whereas lower values reflect offshore feeding (Malpica‐Cruz et al., 2013; Speed et al., 2012). This suggests larger shortfin makos may forage closer to coastal or shelf‐break habitats (Green et al., 2024). Similar size‐related enrichment in δ^13^C values has been observed in tiger sharks in Australia (Ferreira et al., 2017) and Portugal (Maia et al., 2006), interpreted as ontogenetic shifts towards seagrass‐associated or coastal prey. For shortfin makos, this pattern could also reflect regional isotopic baselines associated with productive coastal waters (Young et al., 2010).

δ^34^S values showed no clear trend with size but ranged widely (15.5–22.5‰), overlapping with values typical of food webs relying on benthic epiphytes, seagrass and macroalgae (Belicka et al., 2012; Priester et al., 2024). Mean values clustered around 19‰ across size classes. Similar values have been reported for black marlin (18.9‰), blue marlin (19.3‰) and striped marlin (18.7‰) in the same area (Guillemin et al., 2022), reflecting predominantly pelagic food webs. Similar mean values (17.9–19.3‰) have also been found in two species of sawsharks in the Bass Strait (Burke et al., 2024), supporting the view that values around 19‰ are characteristic of pelagic predators in this region. Although δ^34^S values in shortfin makos spanned a relatively broad range, similar variability has been interpreted as reflecting flexibility in the relative contribution of benthic and pelagic basal resources and habitat use among individuals (Burke et al., 2024). However, because δ^34^S values are typically lower in benthic consumers, these relatively high values suggest that shortfin makos are predominantly pelagic foraging.

Overall, isotope patterns indicate modest ontogenetic shifts in shortfin mako sharks sampled in temperate eastern Australia. Larger individuals displayed slightly lower δ^15^N and larger δ^13^C values, suggesting feeding closer to coastal habitats on prey of lower trophic positions, while δ^34^S values indicate limited benthic influence. However, the nature of our dataset hinders the full and precise interpretation of these isotope values in the context of shortfin mako shark trophic ecology. As with many shark species, obtaining samples across a broad size range is challenging due to difficulties associated with accessing individuals from different life stages. Moreover, almost all males were already mature at the time of sampling and even considering a slow turnover rate in shortfin mako shark muscle tissue of around 2 years, the smallest individuals may have already been sexually mature. Consequently, this dataset represents mainly sub‐adults and adult stages, not capturing potential trophic shifts in juveniles and YOY.

Isotopic niches were built to integrate variation across size and isotopes to assess resource and habitat use. The smallest niche areas and volume were observed in δ^13^C and δ^15^N space, whereas δ^34^S increased niche width due to its broader range of values. Similar niche sizes have been reported for other apex predators in New South Wales. For example, Ferreira et al. (2017) found SEA_c_ values of 1.16–2.06‰^2^ for tiger sharks, while dusky, white and tiger sharks exhibited SEA_c_ values between 0.50 and 1.06‰^2^ (Lipscombe, 2024). These values indicate relatively broad trophic niches, suggesting shortfin mako sharks exploit diverse resources in temperate eastern Australia.

Across isotope pairs, core ellipses overlapped among most size classes, except between the ≤200 cm and >250 cm groups in δ^13^C‐ δ^15^N space. In contrast, the 201–250 cm and >250 cm groups showed higher overlap, sometimes exceeding 50%. Overlap increased further when 95% ellipses were considered; however, these are more sensitive to extreme values (Tamburin et al., 2019). Such overlap patterns have been interpreted as evidence of shared habitats and resources among size classes or co‐occurring predators (Wang et al., 2022). Larger individuals showed less variation in niche space, indicating greater resource and/or habitat sharing (Pinzone et al., 2019), whereas smaller sharks, mainly sub‐adult males and immature females, displayed more variability, suggesting ontogenetic differentiation.

These results indicate an ontogenetic shift in resource use between sub‐adult and adult shortfin mako sharks in temperate eastern Australia. After maturity, sharks of both sexes appear to converge on similar habitats and prey, reflected by increased niche overlap between larger sharks. In other apex predators, including tiger and white sharks, isotopic niches often broaden with size, reflecting more generalized diets (Ferreira et al., 2017; Lipscombe, 2024). Juveniles of several shark species exhibit more specialized feeding behaviour before shifting to generalist strategies as niche width increases (Priester et al., 2024). Similar patterns have been observed in whitespotted eagle rays in eastern Australia, with larger isotopic niches linked to opportunistic feeding (Chan et al., 2022). Isotopic niches have also diverged between pelagic and benthic predators (Riverón Mato, 2022). Pelagic consumers would be considered generalist feeders, exhibiting narrower niches, while individuals relying on inshore resources would display broader niches due to a higher diversity of prey. Overall, shortfin mako isotopic niches suggest ontogenetic shifts in resource use, although additional data are needed to clarify specific feeding strategies during earlier life stages.

### 4.2 Sex and seasonal influence

There is a strong sexual dimorphism in shortfin mako sharks (Preti et al., 2012), with females reaching maturity much later (258 cm FL, 19–21 years) compared to males (179 cm FL, 7–9 years; Stevens, 1983). Moreover, after reaching maturity, males grow at slower rates and might have lower metabolic demands than females. Size was therefore included in the model when testing for sex‐related isotopic differences. Once FL was accounted for, there were no significant sex‐related isotopic differences in *I. oxyrinchus*. Although the largest size group in our dataset was composed almost exclusively of females (23 females and only one male), the adjusted means indicated a tendency for female δ^13^C values to be lower than those of males. It contrasts with the decreasing δ^13^C values observed with size, which would suggest larger females to rely more on inshore benthic food webs, with higher δ^13^C values. In other shark species, mature females have shown high values for carbon isotopic ratios, associated with coastal foraging, like tiger sharks in the north Atlantic (Priester et al., 2024) and sevengill sharks in Tasmania (Abrantes & Barnett, 2011). In Baja California, based on stomach content analysis, male shortfin makos would show a preference for oceanic prey, with females suggested to prefer coastal and benthic zones, and to forage on lower trophic level resources (Velasco‐Tarelo et al., 2024). Therefore, while trophic niches overlap, males and females would have sexual segregation, and males would prefer feeding in oceanic zones with different isotopic baselines. Our study does not provide sufficient evidence regarding such sexual segregation for shortfin mako sharks in eastern Australia. This trend towards lower δ^13^C values, although not significant, might reflect different movement patterns or simply a variability in the baseline in regions frequented by females (Abrantes & Barnett, 2011).

Seasonal differences were evident, with sharks sampled in spring showing higher δ^15^N and lower δ^13^C values than those sampled in summer. When sampling season was included in the linear model, sex became a significant predictor of δ^13^C, with females exhibiting lower δ^13^C values than males. One explanation is that females may move seasonally into regions with different isotopic baselines, such as parturition habitats. Shortfin mako pups are reported to be born off the coast of New South Wales in November (Last & Stevens, 2009), which could lead to females foraging in regions with lower δ^13^C values. However, this hypothesis would apply only to pregnant females, and information on reproductive status was not available in our dataset, so this explanation remains speculative. Shortfin makos are also known to migrate following a clear seasonal cycle with north–south movements in the North Atlantic and Northeast Pacific Oceans, following warm temperatures in winter (Francis et al., 2018).

Interpretation of seasonal patterns must be cautious due to sampling bias. In our dataset, most male samples were collected in spring near Wollongong (south sampling site), while more females were sampled in summer off Port Stephens (north sampling site). This uneven spatial and temporal distribution could amplify or confound seasonal differences. Satellite tagging could help clarify movement patterns and habitat use in the region. Although satellite tagging has already been used for shortfin makos in the Australian region, studies have primarily focused on behaviour an displacement distance, rather than site fidelity (Rogers et al., 2015).

Additional uncertainty arises from the slow isotope turnover rate of isotopes in larger shark muscle, estimated from months to several years but often approximated at about 1 year (Guillemin et al., 2022; Speed et al., 2012). Turnover may also vary with body size, further complicating making its correct estimations (Reum & Essington, 2013). Consequently, isotopic values integrate diet over long and variable periods, meaning the season of sampling may not correspond to the season in which prey were ingested. Seasonal signals therefore likely reflect a combination of migration, sex‐specific movements and sampling biases.

### 4.3 Management and conservation

Our findings may have direct implications for improving the management of *I. oxyrinchus* in temperate eastern Australia. Understanding the trophic role of this species in these waters is crucial for understanding their ecological interactions and for informing effective fisheries management and conservation strategies (Simpfendorfer et al., 2011). The most recent Ecological Risk Assessment for the Eastern Tuna and Billfish Fishery identified makos as being the most vulnerable species despite existing regulatory protections (Sporcic et al., 2019). Over 10,000 shortfin makos were captured between 2011 and 2015, with more than 7000 mortalities. Although commercial fisheries must release live individuals, post‐release mortality may still affect populations (Sporcic et al., 2019). Recreational fisheries also capture large numbers of shortfin makos, historically making them the primary shark species targeted in game fish tournaments (Ghosn et al., 2015).

Stable isotope analysis revealed ontogenetic variation in trophic ecology, suggesting that different life stages may face different fishing pressures. Consistently high δ^15^N values confirm that sub‐adult and adult shortfin makos occupy high trophic positions, indicating an important ecological role in pelagic food webs. Declines in mako abundance could affect ecosystem structure. Higher δ^13^C values in larger individuals, particularly females, suggests increasing use of coastal food webs. If so, mature sharks may interact with inshore recreational fisheries more than previously thought. This pattern aligns with observations of site fidelity to productive continental slope habitats (Green et al., 2024).

These findings support the need for management frameworks that consider both offshore and inshore habitats. Integrating dietary data with trophic roles also contributes to ecosystem‐based fisheries management. Shortfin makos exhibit a generalist strategy at the population level, with possible individual specialization, consistent with patterns in other large predators (Lipscombe, 2024; Priester et al., 2024). Improved knowledge of prey preferences would help predict responses to changes in prey availability and reduce bycatch risk. For example, declines in key prey such as southern calamari could increase predation on commercially valuable teleosts.

Effective management of *I. oxyrinchus* in temperate eastern Australia will therefore require integrating ecological data on trophic role, ontogenetic variation and habitat use with fisheries monitoring (MoU Advisory Committee, 2025). Stable isotope analysis complements tagging programmes, ecological risk assessments and recreational fishing datasets. Together, these approaches can reduce uncertainty in stock assessments and guide conservation under national (EPBC Act) and international (CMS, CITES) frameworks.

## 5 CONCLUSION

This study provides the first stable isotope analysis dedicated to shortfin mako sharks (*Isurus oxyrinchus*) in temperate eastern Australian waters and the first to incorporate sulphur isotopes for the species. Our approach delivered a species‐focused perspective on makos, including ontogenetic variation, isotopic values and niche position dynamics.

While informative, these findings are constrained by several factors that highlight potential directions for future research. Our dataset was relatively small and lacked juvenile and YOY individuals, preventing a full assessment of ontogenetic shifts. Samples were obtained opportunistically from game fishing events, resulting in uneven spatial and temporal coverage. Isotopic turnover rates in shark muscle remain uncertain, complicating interpretation of seasonal and size‐related patterns. In addition, baseline isotopic variation and prey species were not sampled, limiting precise trophic position estimates.

Future research should combine SIA with complementary approaches to improve our understanding of shortfin mako trophic ecology. Key priorities include sampling across life history stages, seasons and locations; analysing tissues with faster turnover rates such as plasma and liver; collecting isotopic data from local prey and other large predators during the same time frame to place shortfin mako sharks in a global trophic framework in the area; complementing SIA with stomach content analyses to directly identify prey; and integrating telemetry or satellite tagging to examine movement patterns and habitat use.

Together, these approaches would provide a more comprehensive understanding of the trophic role of *I. oxyrinchus* in temperate eastern Australia and strengthen the ecological knowledge needed for efficient conservation and fisheries management. Linking ontogenetic variation in diet and habitat use with fisheries interactions will be critical for designing region‐specific, ecosystem‐based management strategies to reduce bycatch and support sustainable populations.

## AUTHOR CONTRIBUTIONS

**Maëlle Devillebichot** contributed to methodology, investigation, data curation, formal analysis, visualization and writing—original draft. **Jane Williamson** contributed to conceptualization, investigation, supervision, funding acquisition, resources and writing—review & editing. **Julian Pepperell** contributed to supervision, funding acquisition, resources and writing—review & editing. **Tristan Guillemin** contributed to conceptualization, investigation, supervision, funding acquisition, formal analysis, resources and writing—review & editing.

## FUNDING INFORMATION

Ocean Health Foundation, School of Natural Sciences at Macquarie University, and Marine Ecology Group at Macquarie University.

## CONFLICT OF INTEREST STATEMENT

The authors declare no conflicts of interest.

## Supporting information


**Table S1.** Results of analysis of variance (ANOVA) II and Tukey's honestly significant difference (HSD) post hoc tests run on linear models to test the effect of sex, FL (cm) and sampling season on the three isotopes values. Significant *p*‐values are in bold; marginal values (0.05 ≤ *p* < 0.1) are followed by a dot.
**Figure S1.** Posterior distributions of Bayesian SEA (SEA_b_) for each size class for the δ13C‐δ15N pair (A), δ13C‐δ34S pair (B) and δ34S‐δ15N pair (C), with credible intervals (50%, 75%, 95%) and SEA_c_ overlaid as white crosses (×).
**Table S2.** Estimated small sample corrected SEA_c_ (‰^2^) for each isotopic pair (δ^13^C‐δ^15^N, δ^13^C‐δ^34^S, δ^34^S–δ^15^N) across shortfin mako size classes (≤200 cm, 201–250 cm, >250 cm), and for all individuals combined (Total SEA_c_).
**Table S3.** Pairwise posterior overlap probabilities (%) between the three size classes in 3D isotopic space, with corresponding SEV_b_ values (‰^3^). Values outside brackets correspond to core niches (ellipses covering 40% of the data), while values in brackets represent total niche space (ellipses covering 95% of the data).

## Data Availability

The underlying data and code supporting the findings of this study are publicly accessible in the open data repository Zenodo under the DOI https://doi.org/10.5281/zenodo.22710532.
