# Supplementary material for: Ontogenetic and spatial variation in the trophic ecology of the shortfin mako shark (Isurus oxyrinchus) in temperate eastern Australia
Source: J Fish Biol. 2026 Sep 27;109(5):2931–45. doi: 10.1111/jfb.70667 (PMC13770694; doi:10.1111/jfb.70667)
Supplement: Supplementary file 1 [file JFB-109-2931-s001.docx]

**Supplementary Material**

**Ontogenetic and spatial variation in the trophic ecology of the shortfin mako shark (*Isurus oxyrinchus*) in temperate eastern Australia**

Maëlle Devillebichot, Tristan Guillemin^1^, Julian Pepperell^2^, Jane E. Williamson^1^

^1^ School of Natural Sciences, Macquarie University, NSW, Australia

^2^ Pepperell Research and consulting Pty Ltd., Noosaville, QLD, Australia

Corresponding author: [jane.williamson@mq.edu.au](mailto:jane.williamson@mq.edu.au)

***Supplementary Table 1****: Results of ANOVA II and Tukey’s HSD post-hoc tests run on linear models to test the effect of sex, FL (cm) and sampling season on the three isotopes values. Significant p-values are in bold; marginal values (0.05 ≤ p < 0.1) are followed by a dot.*

| Response | Parameter | df | F | *p* | Tukey’s HSD |
| --- | --- | --- | --- | --- | --- |
| δ^15^N | Sex | 1 | 1.34 | 0.25 | Spring — Autumn-Winter  **0.03** |
|  | FL | 1 | 11.31 | **0.001** |  |
|  | Season | 2 | 3.41 | **0.04** |  |
| δ^13^C | Sex | 1 | 5.93 | **0.02** | Summer — Autumn-Winter 0.08.  Summer — Spring 0.06. |
|  | FL | 1 | 17.21 | **< 0.001** |  |
|  | Season | 2 | 3.30 | **0.04** |  |
| δ^34^S | Sex | 1 | 0.19 | 0.66 | None |
|  | FL | 1 | 0.22 | 0.64 |  |
|  | Season | 2 | 0.78 | 0.46 |  |

***Supplementary Figure 1****: Posterior distributions of Bayesian SEA (SEA_b_) for each size class for the δ13C-δ15N pair (A), δ13C-δ34S pair (B), and δ34S-δ15N pair (C), with credible intervals (50%, 75%, 95%) and SEA_c_ overlaid as white crosses (×).*

***Supplementary Table 2:*** *Estimated small sample corrected SEA_c_ (‰^2^) for each isotopic pair (δ^13^C-δ^15^N, δ^13^C-δ^34^S, δ^34^S–δ^15^N) across shortfin mako size classes (≤ 200 cm, 201-250 cm, > 250 cm), and for all individuals combined (Total SEA_c_).*

|  | **δ^13^C-δ^15^N** | | | **δ^13^C-δ^34^S** | | | **δ^34^S-δ^15^N** | | |
| --- | --- | --- | --- | --- | --- | --- | --- | --- | --- |
| **Size group** | ≤ 200 | 201-250 | > 250 | ≤ 200 | 201-250 | > 250 | ≤ 200 | 201-250 | > 250 |
| **SEA_c_ (‰^2^)** | 1.29 | 1.16 | 1.33 | 2.30 | 1.69 | 2.27 | 1.94 | 2.10 | 2.82 |
| **Total SEA_c_ (‰^2^)** | 1.63 | | | 2.36 | | | 2.31 | | |

***Supplementary Table 3:*** *Pairwise posterior overlap probabilities (%) between the three size classes in 3D isotopic space, with corresponding SEV_b_values (‰^3^). Values outside brackets correspond to core niches (ellipses covering 40% of the data), while values in brackets represent total niche space (ellipses covering 95% of the data).*

| Size group | ≤  200 | 201-250 | > 250 | SEV_b_ (‰^3^) |
| --- | --- | --- | --- | --- |
| ≤  200 | NA | 23.2 (79.2) | 7.0 (44.9) | 1.24 |
| 210-250 | 17.1 (65.0) | NA | 27.2 (79.0) | 1.61 |
| > 250 | 5.2 (44.5) | 31.4 (86.1) | NA | 1.44 |
| **Total** | — | — | — | 2.07 |
